# Community Assembly of Endophytic Fungi in Ectomycorrhizae of Betulaceae Plants at a Regional Scale

**DOI:** 10.3389/fmicb.2019.03105

**Published:** 2020-01-21

**Authors:** Yong-Long Wang, Cheng Gao, Liang Chen, Niu-Niu Ji, Bin-Wei Wu, Peng-Peng Lü, Xing-Chun Li, Xin Qian, Pulak Maitra, Busayo Joshua Babalola, Yong Zheng, Liang-Dong Guo

**Affiliations:** ^1^State Key Laboratory of Mycology, Institute of Microbiology, Chinese Academy of Sciences, Beijing, China; ^2^College of Life Sciences, University of Chinese Academy of Sciences, Beijing, China; ^3^Key Laboratory of Plant Resources Conservation and Sustainable Utilization, South China Botanical Garden, Chinese Academy of Sciences, Guangzhou, China; ^4^College of Geographical Science, Fujian Normal University, Fuzhou, China

**Keywords:** Betulaceae, dispersal limitation, endophytic fungi, environmental filtering, host phylogeny

## Abstract

The interaction between aboveground and belowground biotic communities drives community assembly of plants and soil microbiota. As an important component of belowground microorganisms, root-associated fungi play pivotal roles in biodiversity maintenance and community assembly of host plants. The Betulaceae plants form ectomycorrhizae with soil fungi and widely distribute in various ecosystems. However, the community assembly of endophytic fungi in ectomycorrhizae is less investigated at a large spatial scale. Here, we examined the endophytic fungal communities in ectomycorrhizae of 22 species in four genera belonging to Betulaceae in Chinese forest ecosystems, using Illumina Miseq sequencing of internal transcribed spacer 2 amplicons. The relative contribution of host phylogeny, climate and soil (environmental filtering) and geographic distance (dispersal limitation) on endophytic fungal community was disentangled. In total, 2,106 endophytic fungal operational taxonomic units (OTUs) were obtained at a 97% sequence similarity level, dominated by Leotiomycetes, Agaricomycetes, Eurotiomycetes, and Sordariomycetes. The endophytic fungal OTU richness was significantly related with host phylogeny, geographic distance, soil and climate. The endophytic fungal community composition was significantly affected by host phylogeny (19.5% of variation explained in fungal community), geographic distance (11.2%), soil (6.1%), and climate (1.4%). This finding suggests that environmental filtering by plant and abiotic variables coupled with dispersal limitation linked to geographic distance determines endophytic fungal community assembly in ectomycorrhizae of Betulaceae plants, with host phylogeny being a stronger determinant than other predictor variables at the regional scale.

## Introduction

The interaction between aboveground and belowground biotic communities drives community dynamics and ecosystem functioning (Hooper et al., 2000; Wardle et al., 2004; Van Dam and Heil, 2011). As an important component of belowground microorganisms, root-associated fungi exhibit a wide range of strategies including mycorrhizal, endophytic, and pathogenic species (Peay et al., 2008), and these fungi can influence plant growth and fitness, plant community dynamics and biogeochemical cycling (Landeweert et al., 2001; van der Heijden et al., 2008; Liang et al., 2016; Almario et al., 2017). Therefore, revealing mechanisms underlying root-associated fungal community assembly will greatly improve our understanding of biodiversity maintenance and community assembly of plants.

Environmental filtering (niche process) and dispersal limitation (neutral process) are two of the fundamental processes in structuring biotic community assembly (Cottenie, 2005). The effect of environmental filtering by host plants (e.g., identity and phylogeny) on root-associated fungal communities has been widely documented in previous studies (e.g., Ishida et al., 2007; Tedersoo et al., 2013; van der Linde et al., 2018; Wu et al., 2018; Schroeder et al., 2019; Sepp et al., 2019; Wang et al., 2019a, b). For example, plant identity significantly shaped root-associated ectomycorrhizal (EM) fungal communities (Ishida et al., 2007; Tedersoo et al., 2012; van der Linde et al., 2018) and arbuscular mycorrhizal (AM) fungal communities (Martinez-Garcia et al., 2015; Ciccolini et al., 2016; Zheng et al., 2016; Sepp et al., 2019) through host specificity, generating diverse substrates and changing microhabitats (Wardle, 2006; Dickie, 2007; Aponte et al., 2010). Likewise, an increasing amount of studies have indicated that host phylogeny strongly predicts root-associated fungal communities, such as total (Wehner et al., 2014; Schroeder et al., 2019), EM (Põlme et al., 2013; Tedersoo et al., 2013; Wu et al., 2018; Wang et al., 2019a), AM (Wang et al., 2019b), pathogenic (Schroeder et al., 2019; Wang et al., 2019b), and saprotrophic (Schroeder et al., 2019) fungi. According to phylogenetic niche conservatism, closely related plants are more similar in morphological and functional traits than distantly related ones (Losos, 2008), and maybe tend to associate with more similar fungal partners.

Environmental filtering by abiotic variables (e.g., soil and climate) has also been found to impact root-associated fungal communities (e.g., Jarvis et al., 2013; Gao et al., 2015; Miyamoto et al., 2015; Mundra et al., 2016; Barnes et al., 2018; Li et al., 2018; Van Geel et al., 2018; Wu et al., 2018; Wang et al., 2019a). For instance, climate differences explained the largest variation of EM fungal community composition in two Japanese mountains (Miyamoto et al., 2015), while soil variables were the most important drivers of AM fungal community at the Europe (Van Geel et al., 2018). In addition, with increasing nitrogen (N) addition, the abundance of fungal species changed (Cox et al., 2010). This could be ascribable to the fact that fungal taxa commonly prefer to certain niche properties and differ in response to environmental changes (Cox et al., 2010; Mcguire et al., 2010; Mundra et al., 2016; Barnes et al., 2018; Miyamoto et al., 2018).

Generally, geographic distance is widely hypothesized to predict biotic community distribution as the stochastic dispersal of individuals from one site to another could be hindered by the spatial distance between sites, suggesting the effect of dispersal limitation on community assembly (Hubbell, 2001). Many previous studies have demonstrated that dispersal limitation strongly determines community assembly of root-associated fungi, such as total fungi (Wehner et al., 2014; Beck et al., 2015), AM fungi (Dumbrell et al., 2010; Davison et al., 2015; Van Geel et al., 2018), and EM fungi (Tedersoo et al., 2012; Põlme et al., 2013; Matsuoka et al., 2016; van der Linde et al., 2018; Wu et al., 2018; Wang et al., 2019a) at various spatial scales and in different ecosystems. Besides the geographic distance, fungal taxa commonly show varying functional traits, such as dispersal, germination, and colonization abilities, which also contribute to dispersal limitation (Nara, 2009; Peay et al., 2012; Kivlin et al., 2014). Additionally, priority effect could also give rise to the variation in fungal community, that is, early-arriving fungal species could colonize much more habitats and resources, and being competitive dominant over the later ones (Kennedy and Bruns, 2005; Kennedy et al., 2009). However, most studies on root-associated fungal community assembly have mainly focused on mycorrhizal fungi (e.g., Põlme et al., 2013; van der Linde et al., 2018; Van Geel et al., 2018; Wu et al., 2018; Sepp et al., 2019; Wang et al., 2019a). In contrast, the community assembly of root endophytic fungi has less been investigated at a large spatial scale.

The Betulaceae plants are typical EM hosts, widely distribute in Chinese forest ecosystems, and have importantly economic and ecological values (Li and Skvortsov, 1999). In addition to EM fungi in ectomycorrhizae, there are also endophytic fungi which can live in plant tissues without causing apparent harm to the host plants (Petrini, 1991). Recently, we have revealed that both environmental filtering (i.e., host phylogeny, soil and climate) and dispersal limitation (i.e., geographic distance) shaped EM fungal community assembly of Betulaceae plants in Chinese forest ecosystems, and environmental filtering by host plant phylogeny was the strongest determinant (Wang et al., 2019a). As previous studies showed that there was high endophytic fungal diversity in ectomycorrhizae of host plants (Gao et al., 2013, 2015; Matsuoka et al., 2016; Wu et al., 2018; Wang et al., 2019a), we analyzed the community assembly of endophytic fungi in ectomycorrhizae associated with Betulaceae plants in this study. We hypothesize that (1) environmental filtering and dispersal limitation affect endophytic fungal community and (2) environmental filtering by host plant phylogeny has a stronger effect on endophytic fungal community than other variables at a large scale.

## Materials and Methods

### Study Site, Sampling, and Molecular Analysis

The detailed description of study site, sampling and molecular analysis can be found in Wang et al. (2019a). Briefly, 300 root samples of 23 plant species belonging to four genera (*Alnus*, *Betula*, *Carpinus*, and *Corylus*) of Betulaceae and 33 rhizosphere soil samples were collected from 26 forest sites in China, extending from 24°30′ N to 52°56′ N, and 94°12′ E to 128°52′ E (Supplementary Figure 1). The geographic coordinates (latitude and longitude) and altitude of each site were recorded using a M-241 GPS instrument (Holux Technology, Inc., Taiwan, China). Mean annual temperature (MAT) and mean annual precipitation (MAP) of each site were compiled from the WorldClim global data set at a resolution of 2.5 min, based on 1970–2000 MAT and MAP (Hijmans et al., 2005). Information on the selected plant species, soil properties, geographical location and climate is available in Wang et al. (2019a).

In total, 44,400 EM root tips from 296 samples (four samples with less than 150 EM root tips were discarded) were used for Illumina MiSeq sequencing of the fungal internal transcribed spacer 2 (ITS2) region of the rDNA (see Wang et al., 2019a). A total of 6,482,896 high-quality ITS2 sequences were obtained from 8,321,293 raw sequences after quality control, and were grouped into 5,763 non-singleton operational taxonomic units (OTUs) at a 97% similarity level. Of these 5,763 OTUs, 4,770 (6,286,352 reads) were identified as fungi based on search against the international nucleotide sequence databases collaboration (INSDC) and the unified system for the DNA-based fungal species linked to the classification (UNITE) databases (Kõljalg et al., 2013) by using the basic local alignment search tool (BLAST) (Altschul et al., 1990). Among the fungal data, 2,667 OTUs (846,012) were endophytic fungi using for subsequent analysis. To eliminate the effect of different sequence numbers on the endophytic fungal community analysis, the number of sequences per sample was normalized to the smallest sample size using the sub. sample command in MOTHUR 1.31.2 (Schloss et al., 2009). The representative endophytic fungal OTU sequences have been submitted to the European Nucleotide Archive (ENA) under study accession nos. LR586058-LR588163. Detailed information about endophytic fungi is summarized in Supplementary Table 1.

### Statistical Analysis

All statistical analyses were conducted in R v.3.5.2 (R Development Core Team, 2018), except for the construction of a plant phylogeny tree. ITS sequences of 22 Betulaceae species (one species was removed as samples of which were discarded after normalization) and *Quercus acutissima* (used as an outgroup) were downloaded from GenBank or sequenced by ourselves (see Wang et al., 2019a). The plant sequences were firstly aligned using the L-INS-i algorithm in MAFFT v. 7. 215 (Katoh and Standley, 2013), and then used to construct a maximum likelihood phylogenetic tree by adopting a general time reversible model with 1000 bootstrap replicates in MEGA ver. 7.0 (Kumar et al., 2016) (Supplementary Figure 2). Pairwise patristic distances (pairwise sums of the branch lengths connecting two terminal taxa) were calculated based on the phylogenetic tree by using the cophenetic. phylo command in the ape package (Paradis et al., 2004). Principal coordinates analysis (PCoA) was adopted to transform the pairwise patristic distance matrix to phylogenetic eigenvectors by applying the cmdscale command in the vegan package (Oksanen et al., 2013). Spatial eigenvectors of principal coordinate of neighbor matrices (PCNMs) were constructed based on the transformation of geographic distance (latitude and longitude) and only those with positive eigenvalues were retained by using the pcnm command in the PCNM package (Dray et al., 2006). Significant host phylogenetic PCoA and spatial PCNM vectors were forward-selected prior to subsequent statistical analyses using the forward. sel command in the packfor package (Dray et al., 2009).

Rarefaction curves depicting endophytic fungal OTU numbers in each plant genus were obtained by using the specaccum command in the vegan package (Oksanen et al., 2013). The endophytic fungal OTU richness and relative abundances of four most abundant endophytic fungal classes (each > 9% of total endophytic fungal reads) did not satisfy the normality of distribution and homogeneity of variance among the four plant genera before and after logarithmic and square root transformation. Thus, Kruskal–Wallis tests were adopted to examine the effect of plant genus on the endophytic fungal OTU richness and relative abundances of the four abundant endophytic fungal classes. Then Dunn’s tests with Bonferroni adjustment were carried out to detect the significant differences in these respects among the four plant genera using the *post hoc*.kruskal.dunn. test function in the PMCMR package (Pohlert, 2014). Generalized linear models (GLMs) with Poisson distribution and log link function were constructed to determine the effect of spatial, host, soil and climatic variables on endophytic fungal OTU richness. After fitting the global model, the dredge command in the MuMIn package (Bartón, 2018) was firstly used to build all possible candidate models those derived from the different combinations of variables in the global model. Because the response variable was over-dispersed in the models, QAICc (quasi-likelihood adjusted Akaike Information Criterion) was used for the model selection. In order to account for the uncertainty in model selection, model averaging method was applied based on candidate models within 2 ΔQAICc units from the model with the lowest QAICc value by using the model. avg command in the MuMIn package (Bartón, 2018). The relative importance of each variable was generated by summing the QAICc weights from candidate models where they appeared. The variables with significant relative importance in the averaged model were regarded as the best predictors for endophytic fungal OTU richness.

A distance matrix of endophytic fungal community (Hellinger transformed OTU read data) was constructed by calculating dissimilarities using the Bray–Curtis method (Clarke et al., 2006). Endophytic fungal community dissimilarities were visualized by PCoA ordination using the pcoa command in the ape package (Paradis et al., 2004), and the ellipses reflecting 95% confidence intervals were draw around clusters using the ordiellipse function in the vegan package (Oksanen et al., 2013). Meanwhile, analysis of similarity (ANOSIM) was applied to assess the significance of difference in fungal community composition among four plant genera by using the anosim command in the vegan package (Oksanen et al., 2013). Permutational multivariate analysis of variance (PerMANOVA) was implemented to determine the relative importance of host, spatial, soil and climatic variables on endophytic fungal community composition using the adonis command in the vegan package (Oksanen et al., 2013). We also adopted variation partitioning in redundancy analysis to quantify the contribution of host, spatial, soil and climatic variables to the endophytic fungal community richness and composition by using the varpart command in the vegan package (Oksanen et al., 2013).

In order to eliminate the influence of sites on assessing the plant/fungus preferences, two sites (i.e., Changbaishan and Taiyueshan) with the number of plant species ≥ 4 were selected for the preference analysis according to Toju et al. (2016). Additionally, we also calculated index $H_{2}^{\prime }$ characterizing interaction specialization at community level and checkerboard scores reflecting co-occurrences of fungal species within community (see Wang et al., 2019a).

## Results

### Identification of Endophytic Fungi

A total of 2,667 (846,012 reads) endophytic fungal OTUs was obtained from 4,770 (6,286,352 reads) fungal OTUs at 97% sequence similarity level. As the number of endophytic fungal sequences varied from 30 to 12,791 across all samples, a random re-sampling procedure was implemented to construct a subset to a depth of 500 sequences per sample (46 samples with reads less than 500 were excluded), resulting a normalized dataset containing 2,106 endophytic fungal OTUs across 250 samples. The 100 most abundant OTUs accounted for 66.7% of the total endophytic fungal reads (Supplementary Figure 3A). The frequency distribution of endophytic fungal OTUs had a long tail, with 1,842 OTUs occurring in no more than 10 samples (Supplementary Figure 3B). However, the rarefaction curves for each of four plant genera did not reach plateau (Supplementary Figure 4).

### Endophytic Fungal OTU Richness

**FIGURE 1:**
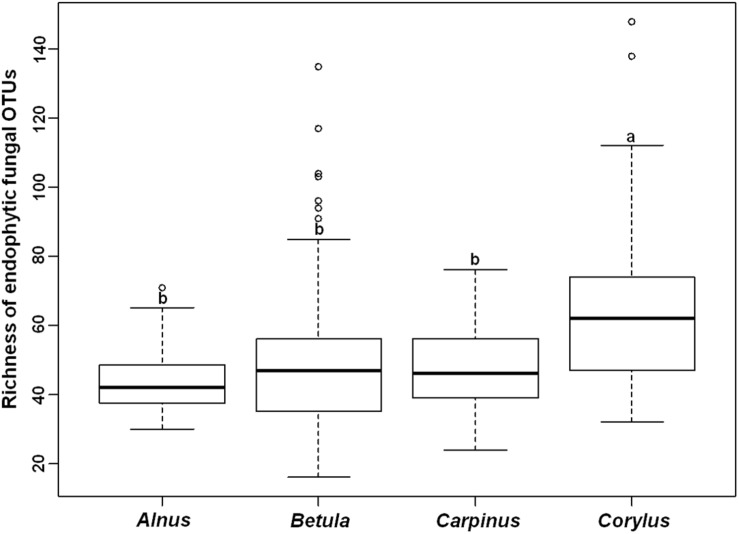
Richness of endophytic fungal operational taxonomic units (OTUs) in four plant genera. Bars without shared letters indicate significant differences in richness of fungal OTUs among the four host genera according to Dunn’s tests with Bonferroni adjustment at *P* < 0.05.

**FIGURE 2:**
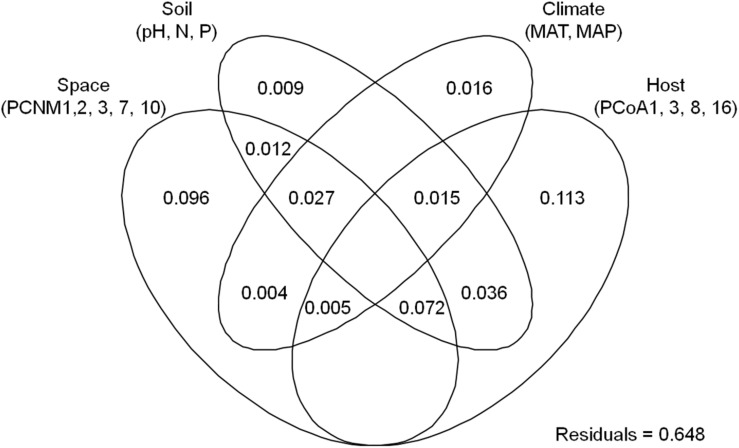
Result of variation partitioning analysis showing pure and shared effects of plant, soil, climatic and spatial variables on endophytic fungal richness. Numbers indicate proportions of explained variation (adjusted *R*^2^ values). PCNM, spatial principal coordinates of neighbor matrices; PCoA, principal coordinates analysis of host phylogeny; N, soil total nitrogen; P, soil total phosphorus; MAT, mean annual temperature; MAP, mean annual precipitation.

The endophytic fungal OTU richness was 44.3 ± 2.2 in *Alnus*, 48.1 ± 1.6 in *Betula*, 47.9 ± 2.1 in *Carpinus*, and 64.8 ± 4.1 (mean ± SE) in *Corylus*, respectively. Kruskal–Wallis test indicated that plant genus significantly affected the OTU richness of endophytic fungi (χ^2^ = 21.048, *P* < 0.001). For example, endophytic fungal OTU richness was significantly higher in *Corylus* than in other three plant genera, but no significant difference was observed in the three plant genera (Figure 1). The final averaged model based on 11 candidate models, suggested that host phylogeny (PCoA1, PCoA3, PCoA8, and PCoA16), spatial vectors (PCNM1, PCNM2, PCNM3, PCNM7, and PCNM10), MAT and soil total N were the best predictor variables for endophytic fungal OTU richness (Table 1 and Supplementary Table 2). In addition, variation partitioning showed that 35.2% of variation in endophytic fungal OTU richness was explained by host phylogeny (19.0%), spatial distance (18.1%), soil (16.5%) and climate (4.4%), with pure effects of 11.3, 9.6, 0.9, and 1.6%, respectively (Figure 2).

**TABLE 1:** Plant and abiotic predictor variables for the richness of endophytic fungal operational taxonomic units following the model-averaging procedure.

**Variable**	**Estimate**	***SE***	***z*-Value**	***P*-value**	**Relative importance**	**Models containing variable**
(Intercept)	4.01E + 00	6.25E-02	64.160	<0.001		
PCoA1	−1.99E + 00	2.73E-01	7.281	<0.001	1.00	11
PCoA3	−7.70E + 00	9.87E-01	7.765	<0.001	1.00	11
PCoA8	−1.89E + 01	2.84E + 00	6.636	<0.001	1.00	11
PCoA16	−5.09E + 09	5.02E + 08	10.101	<0.001	1.00	11
PCNM1	6.61E-08	1.38E-08	4.784	<0.001	1.00	11
PCNM2	−3.31E-08	1.22E-08	2.705	0.007	0.30	3
PCNM3	8.83E-08	9.52E-09	9.236	<0.001	1.00	11
PCNM7	7.40E-08	1.64E-08	4.490	<0.001	1.00	11
PCNM10	−2.05E-07	3.80E-08	5.389	<0.001	1.00	11
MAT	−1.56E-02	4.61E-03	3.366	0.001	0.93	10
Total N	−8.08E-02	3.99E-02	2.017	0.044	0.17	2

*The values shown are the model-averaged coefficients. PCoA, principal coordinates analysis of host phylogeny; PCNM, spatial principal coordinates of neighbor matrices; MAT, mean annual temperature; N, soil nitrogen.*

### Endophytic Fungal Community Composition

**FIGURE 3:**
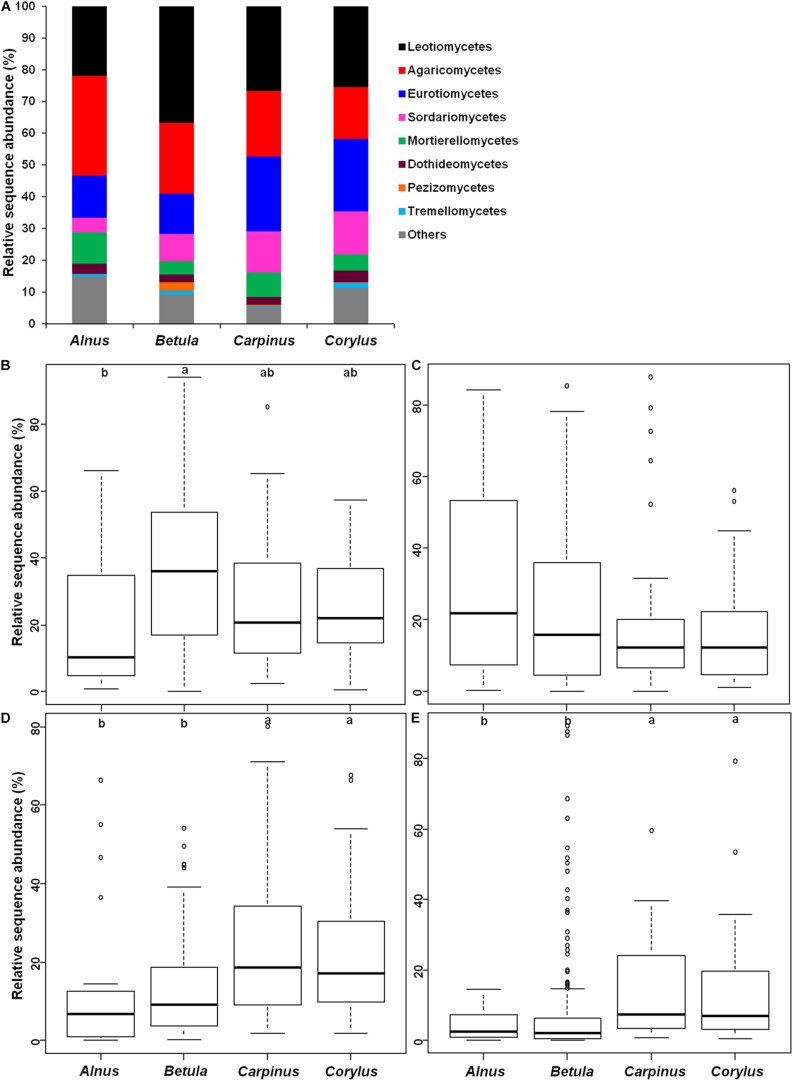
Relative abundance of endophytic fungal classes in four plant genera. **(A)** Abundant classes (>1%) and others (unclassified and rare classes), **(B)** Leotiomycetes, **(C)** Agaricomycetes, **(D)** Eurotiomycetes, and **(E)** Sordariomycetes. Bars without shared letters indicate significant differences according to Dunn’s tests with Bonferroni adjustment at *P* < 0.05.

The endophytic fungal community was dominated by Leotiomycetes, Agaricomycetes, Eurotiomycetes, and Sordariomycetes (Figure 3A). Kruskal–Wallis tests showed that plant genus had a significant effect on the relative abundance of Leotiomycetes (χ^2^ = 15.829, *P* = 0.001), Eurotiomycetes (χ^2^ = 23.32, *P* < 0.001) and Sordariomycetes (χ^2^ = 28.773, *P* < 0.001), but not Agaricomycetes (χ^2^ = 3.428, *P* = 0.33) (Figures 3B–E). For example, the relative abundance of Leotiomycetes was significantly higher in *Betula* than in *Alnus* (Figure 3B). The relative abundances of Eurotiomycetes and Sordariomycetes were significantly lower in *Alnus* and *Betula* than in *Carpinus* and *Corylus* (Figures 3D,E).

**FIGURE 4:**
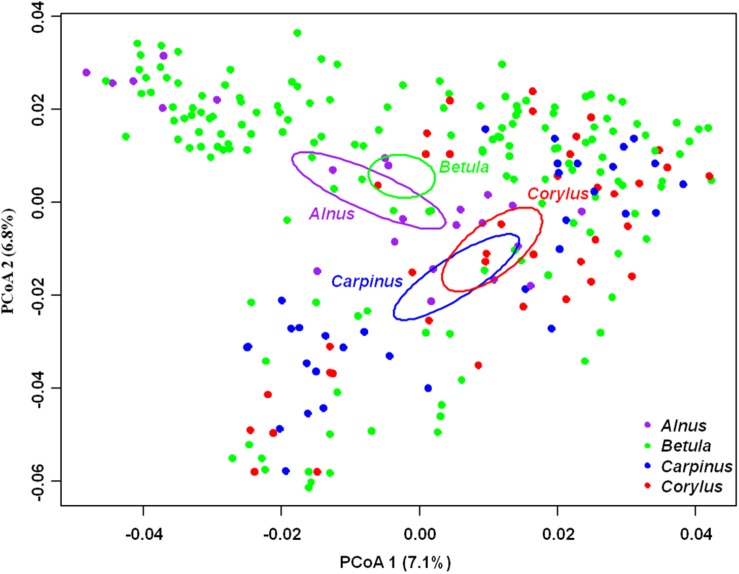
Principal coordinates analysis (PCoA) of endophytic fungal community composition. Percentage of variance explained by each PCoA axis is shown in parenthesis.

**FIGURE 5:**
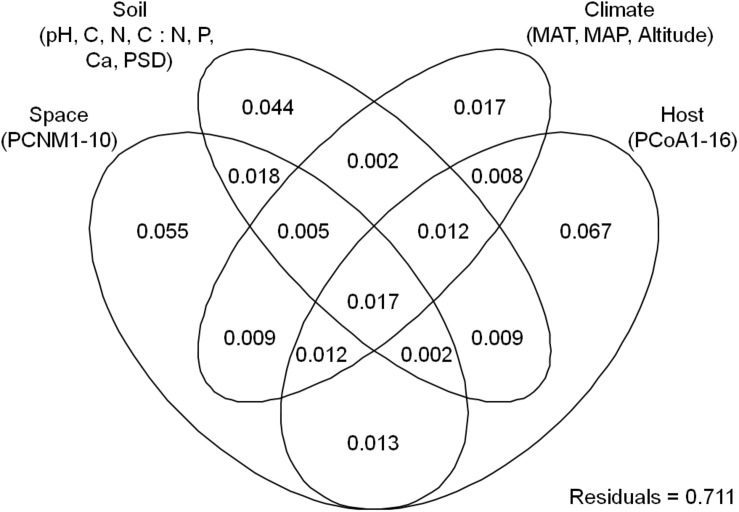
Result of variation partitioning analysis showing pure and shared effects of plant, soil, climatic and spatial variables on endophytic fungal community composition. Numbers indicate proportions of explained variation (adjusted *R*^2^ values). PCNM, spatial principal coordinates of neighbor matrices; PCoA, principal coordinates analysis of host phylogeny; C, soil total carbon; N, soil total nitrogen; P, soil total phosphorus; Ca, soil total calcium; PSD, soil particle size distribution; MAT, mean annual temperature; MAP, mean annual precipitation.

The PCoA ordination indicated that the endophytic fungal community composition was significantly different across the four plant genera (ANOSIM, *R* = 0.11, *P* = 0.001), with the first two principle coordinates explaining 7.1 and 6.8% of variation in fungal community, respectively (Figure 4). PerMANOVA revealed that 39.2% of variation in endophytic fungal community composition was explained, in which host phylogeny (PCoA1-16) explained the most (19.5%), followed by spatial vectors (PCNM1-10; 11.2%), soil (6.1%), climate (1.4%) and altitude (0.9%) (Table 2). Similarly, variation partitioning demonstrated that 28.9% of variation in endophytic fungal community composition was explained by host phylogeny (14.0%), spatial vectors (13.1%), soil (10.8%) and climate (8.1%), with pure effects of 6.7, 5.5, 4.4, and 1.7%, respectively (Figure 5). Taken together, environmental filtering coupled with dispersal limitation drives endophytic fungal community assembly, with a stronger effect of host phylogeny than that of other variables.

**TABLE 2:** Relative importance of plant and abiotic variables on endophytic fungal community composition revealed by Permutational multivariate analysis of variance.

**Variable**	**df**	**Deviance**	***F*-statistics**	***R*^2^**	***P*-value**
Host phylogeny	16	20.370	4.267	0.195	0.001
Spatial vector	10	11.723	3.929	0.112	0.001
Soil pH	1	0.993	3.329	0.010	0.001
Total C	1	1.370	4.593	0.013	0.001
Total N	1	0.940	3.152	0.009	0.001
C: N ration	1	1.184	3.969	0.011	0.001
Total Ca	1	0.652	2.186	0.006	0.001
Total P	1	0.626	2.098	0.006	0.001
PSD	1	0.664	2.226	0.006	0.001
MAT	1	0.847	2.839	0.008	0.001
MAP	1	0.614	2.056	0.006	0.001
Altitude	1	0.941	3.155	0.009	0.001
Residuals	214	63.558		0.608	
Total	250	104.484		1	

*C, soil carbon; N, soil nitrogen; P, soil phosphorus; Ca, soil calcium; PSD, soil particle size distribution; MAT, mean annual temperature; MAP, mean annual precipitation.*

### Host/Fungus Preference and Checkerboard Score

**FIGURE 6:**
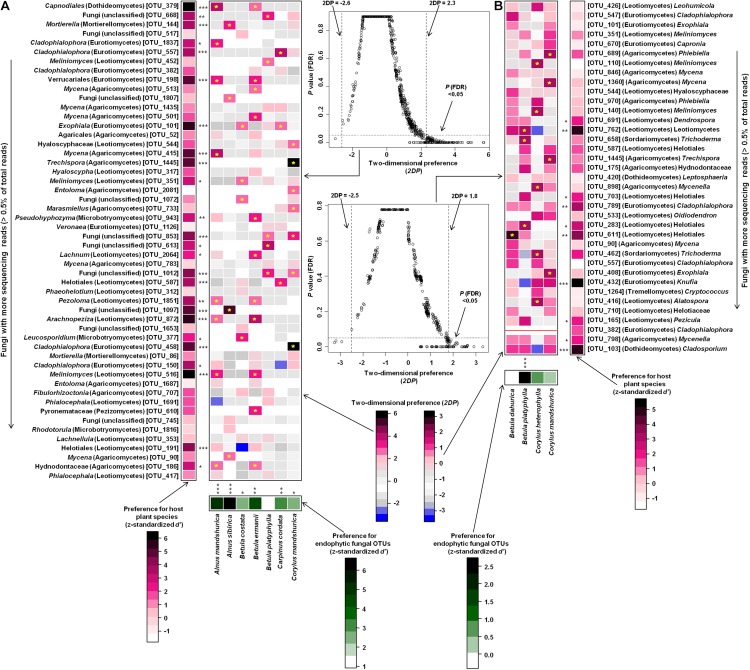
Preferences observed in plant and endophytic fungus associations in Changbaishan **(A)** and Taiyueshan **(B)**. Standardized *d*′ estimates of preferences for fungal OTUs for indicated plant species (columns). Likewise, the standardized *d*′ estimate of preferences for plant species is indicated for each of the observed fungal OTUs (row). A cell in the matrix indicates a two-dimensional preference (*2DP*) estimate, indicating the extent an association of a focal plant–fungus pair was observed more/less frequently than expected by chance. The cell with asterisk inside represents significant preferences in plant–fungus pair. The relationship between *2DP* and FDR-adjusted *P*-values shows that *2DP* values larger than 2.3 (Changbaishan) and 1.8 (Taiyueshan) and those smaller than –2.6 (Changbaishan) and –2.5 (Taiyueshan) represented strong preferences and avoidance, respectively. Red line indicates that the *2DP* value is not available as the standard deviation of the number of samples for the focal plant–fungal OTU pair across randomized matrices is zero. Because multiple species/OTUs were tested, the *P* values are shown as false discovery rates (FDRs) in the plant/fungus analysis. ^∗^*P* < 0.05, ^∗∗^*P* < 0.01, ^∗∗∗^*P* < 0.001.

**FIGURE 7:**
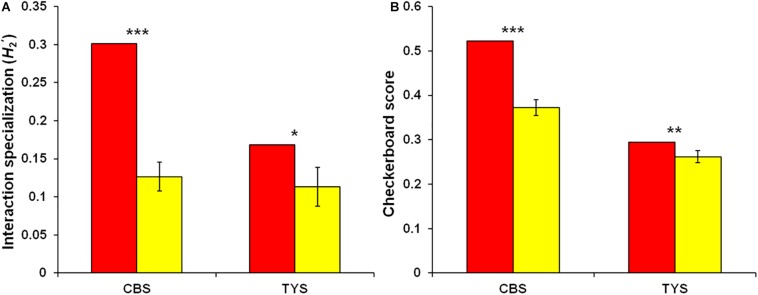
Community-level interaction specialization and checkerboard score of endophytic fungal community. **(A)** $H_{2}^{\prime }$ metric and **(B)** Checkerboard score reflecting co-occurrence within fungal community. The significant difference was estimated by comparing the observed value (red) with randomized values (yellow; mean ± SD) based on two-tailed *t*-test. ^∗^*P* < 0.05, ^∗∗^*P* < 0.01, ^∗∗∗^*P* < 0.001.

Host/fungus preference analysis performed in Changbaishan site indicated that six of seven plant species showed significant preferences for abundant fungi (>0.5% of total sequences), 25 of 52 abundant fungal OTUs showed significant preferences for host plants, and 42 pairs of plants and abundant fungi exhibited remarkably strong preferences (Figure 6A). In Taiyueshan site, one of four plant species and 10 of 37 abundant fungi showed significant preferences toward partners, and 13 pairs of plants and abundant fungi indicated remarkably strong preferences (Figure 6B). In addition, the plant–fungus associations in both sites were significantly specialized at community level and there was less co-occurrence within endophytic fungal communities, as the observed values were all significantly higher than that generated by randomized null models (Figures 7A,B).

## Discussion

We found that the endophytic fungal OTU richness was significantly higher in *Corylus* than in other three plant genera. Furthermore, the endophytic fungal OTU richness was affected by host phylogeny, geographic distance, MAT and soil total N. Similar results were reported in previous studies on root-associated fungal communities, such as EM fungi (Tedersoo et al., 2013; Wu et al., 2018; Wang et al., 2019a), AM fungi (Davison et al., 2015; Van Geel et al., 2018) and pathogenic fungi (Wang et al., 2019b).

We found that host phylogeny significantly shaped endophytic fungal community composition. This finding suggests the important role of environmental filtering derived from host plants in determining fungal communities. All host phylogenetic variables (PCoA1-16) were retained as drivers for endophytic fungal community assembly in present study, suggesting that the splits among host plants at various taxonomic levels could affect fungal community (Tedersoo et al., 2013; Wu et al., 2018; Wang et al., 2019a). The host effect can be explained by phylogenetic niche conservatism, namely, closely related host species are more morphologically and functionally similar than distantly related species (Losos, 2008), and tend to share more similar fungal community. Indeed, in present study the distribution pattern of endophytic fungal community in the ordination corresponded well with phylogenetic position of the four plant genera in the phylogenetic tree. Namely, plant phylogenetic tree demonstrated that *Alnus* and *Betula* were relatively closely related genera, whilst *Carpinus* and *Corylus* were another two closely related genera (Supplementary Figure 2). Meanwhile, the PCoA ordination showed a relatively close cluster of endophytic fungal communities in *Alnus* and *Betula*, whilst *Carpinus* and *Corylus* exhibited another one (Figure 4). Additionally, the specificity between plants and fungi that derived from their long history of co-evolution has been widely documented to influence root-associated fungal communities including EM fungi (Tedersoo et al., 2010; Wu et al., 2018; Wang et al., 2019a), AM fungi (Sepp et al., 2019), endophytic fungi (Maciá-Vicente et al., 2008; Luo et al., 2017) and total fungi (Toju et al., 2016; Schroeder et al., 2019). Indeed, our plant/fungus preference analysis conducted in two sites showed that some abundant endophytic fungi and plant species significantly preferred to some certain partners. Moreover, community-level analyses in both sites also suggested that significantly specificity existed in plant–fungus associations in present study.

In addition to environmental filtering by host plants, abiotic environmental variables including soil (pH, nutrients, and particle size distribution) and climate (MAT and MAP) have also been observed to affect endophytic fungal community in this study. Similar findings were also reported in some studies on root-associated fungal communities, such as endophytic fungi (Maciá-Vicente et al., 2012; Glynou et al., 2016), EM fungi (Jarvis et al., 2013; Miyamoto et al., 2015; Wang et al., 2019a), AM fungi (Dumbrell et al., 2010; Zheng et al., 2014; Van Geel et al., 2018), pathogenic fungi (Wang et al., 2019b), and total fungi (Barnes et al., 2018; Li et al., 2018). Fungal taxa are known to prefer to different niche properties including nutrients and climate (Cox et al., 2010; Mcguire et al., 2010; Mundra et al., 2016; Barnes et al., 2018; Miyamoto et al., 2018). For example, some fungal taxa preferred to nutrient-rich habitats, while some taxa could grow well in nutrient-poor habitats (Cox et al., 2010; Mundra et al., 2016). In addition, some fungi only distributed in habitats with lower MAT and exhibited narrower temperature breadths, while some fungi were detected across wider temperature ranges and in warmer habitats (Miyamoto et al., 2018).

The endophytic fungal community composition was also affected by geographic distance, which is in accordance with the community assembly of other root-associated fungi, such as EM fungi (Tedersoo et al., 2011; Matsuoka et al., 2016; Wang et al., 2019a), AM fungi (Dumbrell et al., 2010; Wang et al., 2019b), pathogenetic fungi (Schroeder et al., 2019; Wang et al., 2019b), and total fungi (Wehner et al., 2014; Beck et al., 2015; Schroeder et al., 2019). All spatial vectors (PCNM1-10) were retained as predictors for endophytic fungal community composition in this study, reflecting fungal community assembly at various spatial variation ranging from relatively small to large scales (Tedersoo et al., 2011; Wu et al., 2018; Wang et al., 2019a). This pattern suggests that dispersal limitation is an important driver of endophytic fungal community turnover. The dispersal limitation effect could be attributable to the geographic isolation generating dispersal barriers that hamper the movement of fungal propagules (e.g., spore and hyphae) from the original to new habitats (Peay et al., 2010, 2012). In addition, fungal taxa *per se* commonly differ in their dispersal abilities, and the order of fungal propagules arriving new habitats could influence the outcome of fungal competition, and then cause fungal community variation (Kennedy and Bruns, 2005; Kennedy et al., 2009). Indeed, our checkerboard score analysis indicated that endophytic fungal community was competitively structured. This is in agreement with previous findings in root-associated fungal communities, such as EM fungi (Pickles et al., 2012; Wu et al., 2018; Wang et al., 2019a) and total fungi (Wehner et al., 2014; Toju et al., 2016).

Additionally, we found that host phylogeny showed a stronger effect on endophytic fungal community than other predictor variables tested in present study. This finding coincides with previous studies concerning root-associated fungi involved in a wide phylogenetic spectra of plants (17 to 51 species of two to 39 genera belonging to one to 22 families) (Tedersoo et al., 2013; Wehner et al., 2014; Wu et al., 2018; Schroeder et al., 2019; Wang et al., 2019a, b), yet conflicts with studies only considered a set of closely related plant species (fewer than seven species in one genus) (Tedersoo et al., 2009; Glassman et al., 2015, 2017; Erlandson et al., 2016). For example, host phylogeny explained more variation of fungal community than spatial and soil variables in studies involving in 25 species of 22 genera in Asteraceae (Wehner et al., 2014) and 45 plant species in 39 genera from 22 families (Wang et al., 2019b). By contrast, in studies those only considering less phylogenetically distant host plants (<7 species in one genus) (Tedersoo et al., 2009; Glassman et al., 2015, 2017; Erlandson et al., 2016), geographic distance and/or abiotic environmental variables showed stronger effects than host plants in determining fungal community composition. In our study, 22 plant species in four genera belonging to Betulaceae represented a relatively wide phylogenetic range and resulted in a stronger host effect relative to other variables on fungal community assembly. These studies suggest that host effect on fungal community assembly is phylogenetic scale-dependent.

We found that some endophytic fungal taxa obtained in present study could be classified as saprophytic and pathogenic groups in previous studies. For example, *Cladophialophora*, *Mycena*, *Mortierella*, and *Meliniomyces* could be assigned to saprophytic fungi, and *Fusarium* and *Neonectria* have been identified as plant pathogenic fungi (Rossman and Palm-Hernández, 2008; Tedersoo et al., 2014; Voříšková et al., 2014). Additionally, members of *Phialocephala*, as globally distributed root endophytic fungal taxa, have been proven to harbor a broad gene inventory that links pathogenic and saprophytic lifestyles (Schlegel et al., 2016). Although some endophytic fungal OTUs in our study showed latent saprophytic or pathogenic functions as reported in previous studies, the ectomycorrhizae selected for analysis in present study were all apparently healthy root tips without any disease. This finding suggests that endophytic fungi could include some potential saprophytic and pathogenic fungi, and their function need to be explored in future research. In conclusion, this study firstly characterizes endophytic fungal community assembly in the ectomycorrhizae of Betulaceae plants at a large scale (*ca*. 2.3 million km^2^). The endophytic fungal OTU richness was significantly related with host phylogeny, geographic distance, soil, and climate. The endophytic fungal community composition was significantly influenced by host phylogeny, geographic distance, soil and climatic variables, in which host phylogeny explained a larger variation than other predictor variables. This finding suggests that both environmental filtering (i.e., host plant phylogeny, soil and climate) and dispersal limitation (i.e., geographic distance) significantly drive community turnover of endophytic fungi, with host phylogeny being a better predictor than any other variables. Our study contributes to a growing literature showing the effect of environmental filtering and dispersal limitation on fungal community.

## Data Availability Statement

The datasets generated for this study can be found in the European Nucleotide Archive, LR586058–LR588163.

## Author Contributions

Y-LW and L-DG conceived and designed the study. Y-LW performed the experiments, analyzed the data, and drafted the manuscript. Y-LW, CG, LC, N-NJ, B-WW, P-PL, X-CL, and YZ performed the field sampling work. XQ, PM, and BB gave some suggestion for English improvement. L-DG revised and approved the final manuscript.

## Conflict of Interest

The authors declare that the research was conducted in the absence of any commercial or financial relationships that could be construed as a potential conflict of interest.
