# Supplementary material for: Community Assembly of Endophytic Fungi in Ectomycorrhizae of Betulaceae Plants at a Regional Scale
Source: Front Microbiol. 2020 Jan 21;10:3105. doi: 10.3389/fmicb.2019.03105 (PMC6986194; doi:10.3389/fmicb.2019.03105)
Supplement: Supplementary file 2 [file Data_Sheet_1.docx]

Supplementary Materials for

**Community Assembly of Endophytic Fungi in Ectomycorrhizae of Betulaceae plants at a Regional Scale**

**Long-Yong W****ang, Cheng Gao, Liang Chen, Niu-Niu Ji, Wei-Bin Wu, Peng-Peng Lü, Xing-Chun Li, Xin Qian, Pulak Maitra, Busayo Joshua Babalola, Yong Zheng and Liang-Dong Guo**

*** Correspondence:** Liang-Dong Guo: *guold@im.ac.cn*

# Supplementary Figures and Tables

## Supplementary Figures


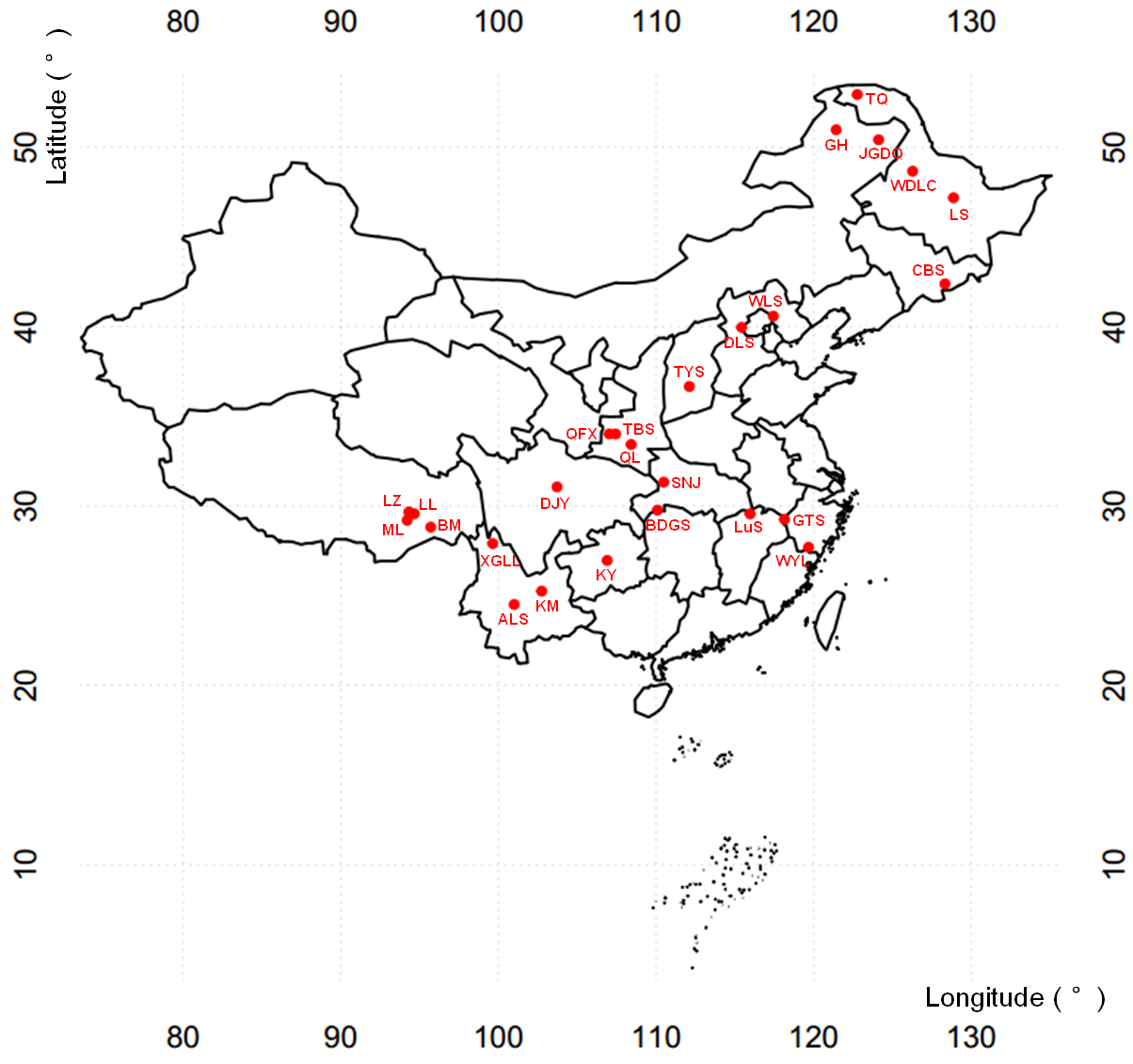


**Supplementary Figure 1.** Sampling sites in secondary forest ecosystems in China. GH, Genhe; JGDQ, Jiagedaqi; TQ, Tuqiang; WDLC, Wudalianchi; LS, Liangshui; CBS, Changbaishan; WLS, Wulingshan; DLS, Donglingshan; TYS, Taiyueshan; TBS, Taibaishan; QL, Qinling; QFX, Qingfengxia; SNJ, Shennongjia; DJY, Dujiangyan; LuS, Lushan; GTS, Gutianshan; WYL, Wuyanling; BDG, Badagong; KY, Kaiyang; KM, Kunming; ALS, Ailaoshan; XGLL, Xianggelila; LZ, Linzhi; ML, Milin; LL, Lulang; BM, Bomi.


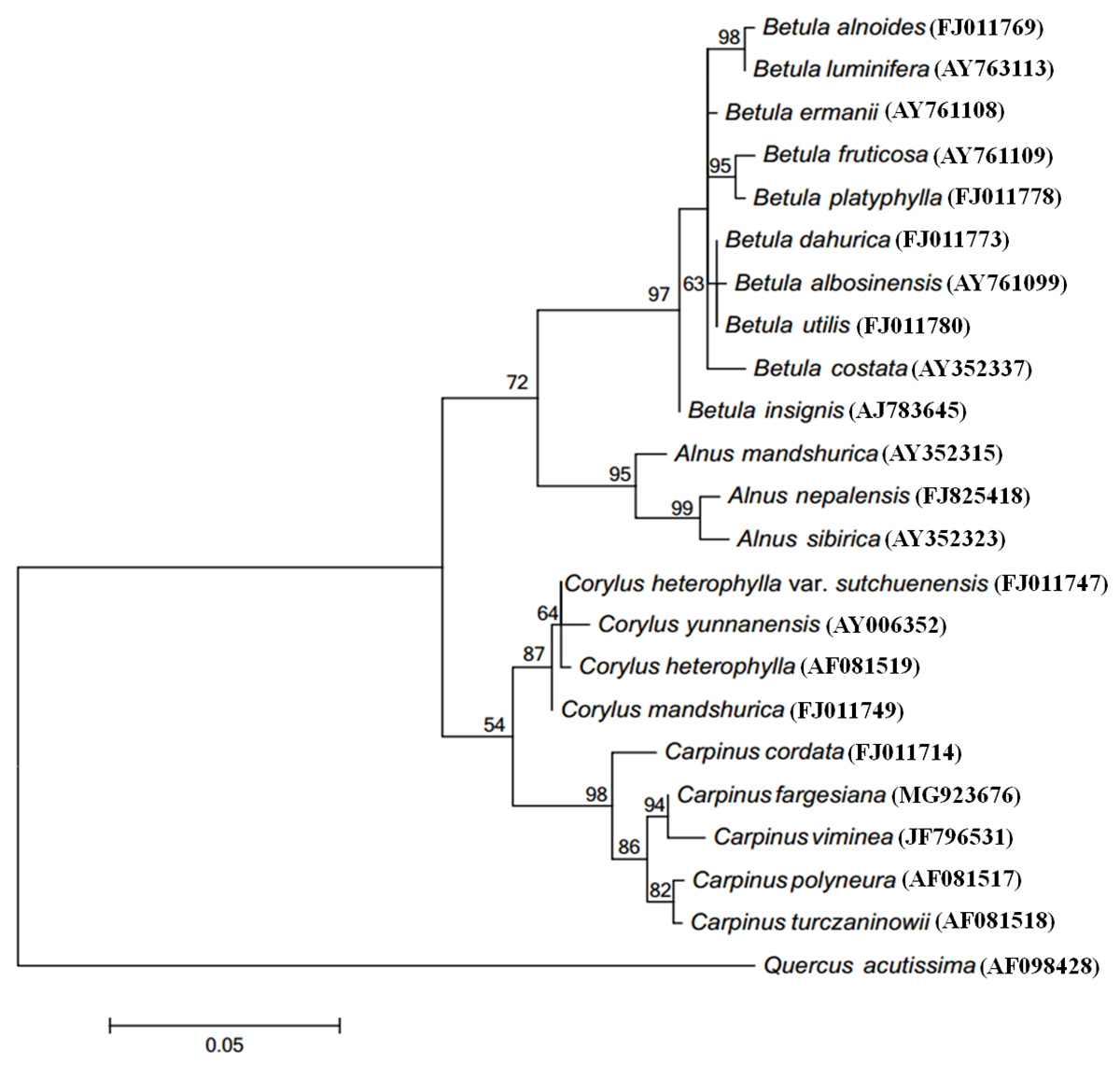


**Supplementary Figure 2.** Maximum likelihood phylogram demonstrating phylogenetic placement of Betulaceae plants based on internal transcribed spacer sequences, using *Quercu acutissima* as an outgroup. The corresponding accession numbers are indicated in parentheses. Bootstrap support values ≥ 50% are shown on branches. Scale bar represents 5% sequence divergence.


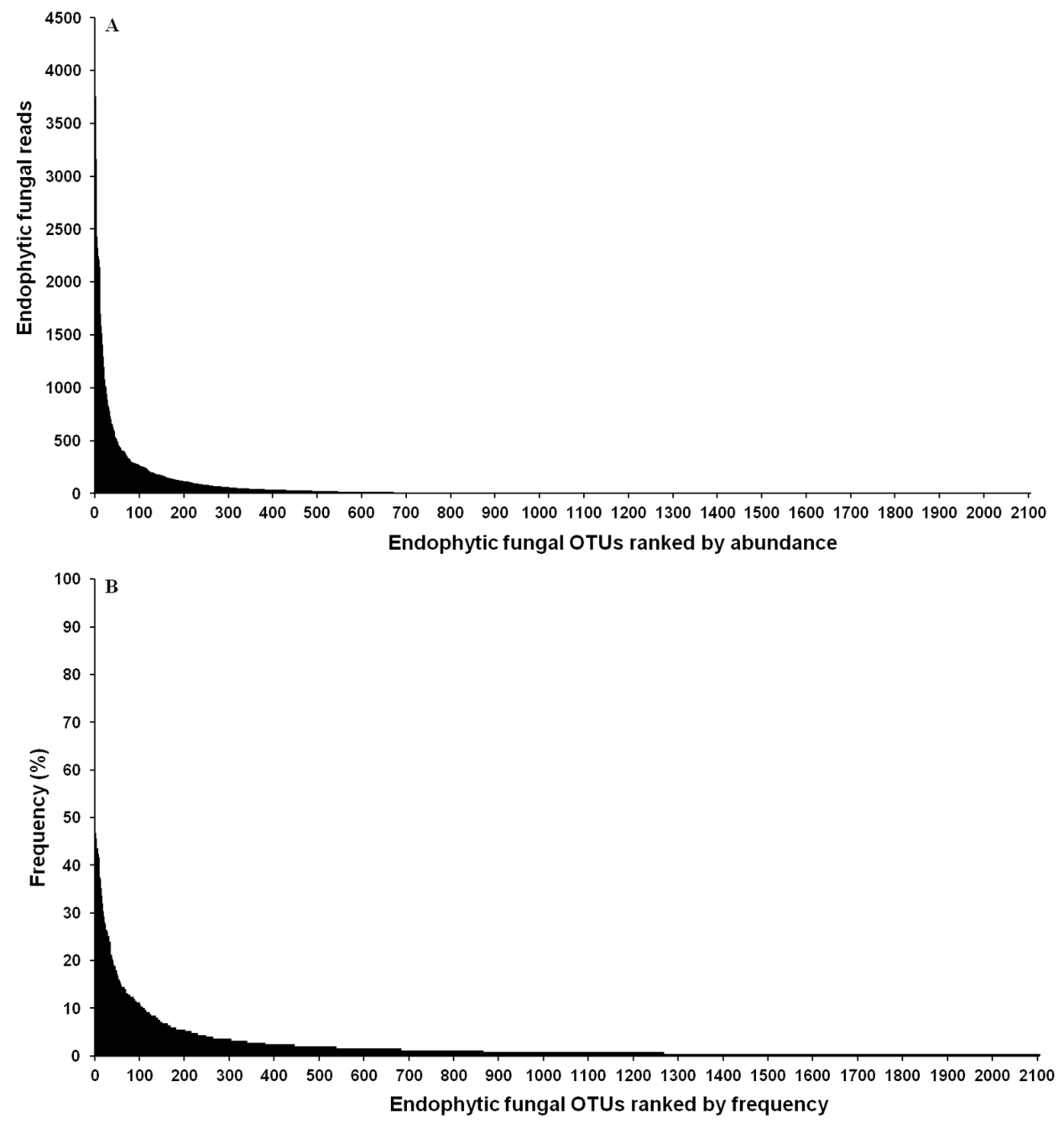


**Supplementary Figure 3.** Endophytic fungal operational taxonomic units (OTUs) ranked by abundance **(A)** and frequency **(B)**.

**
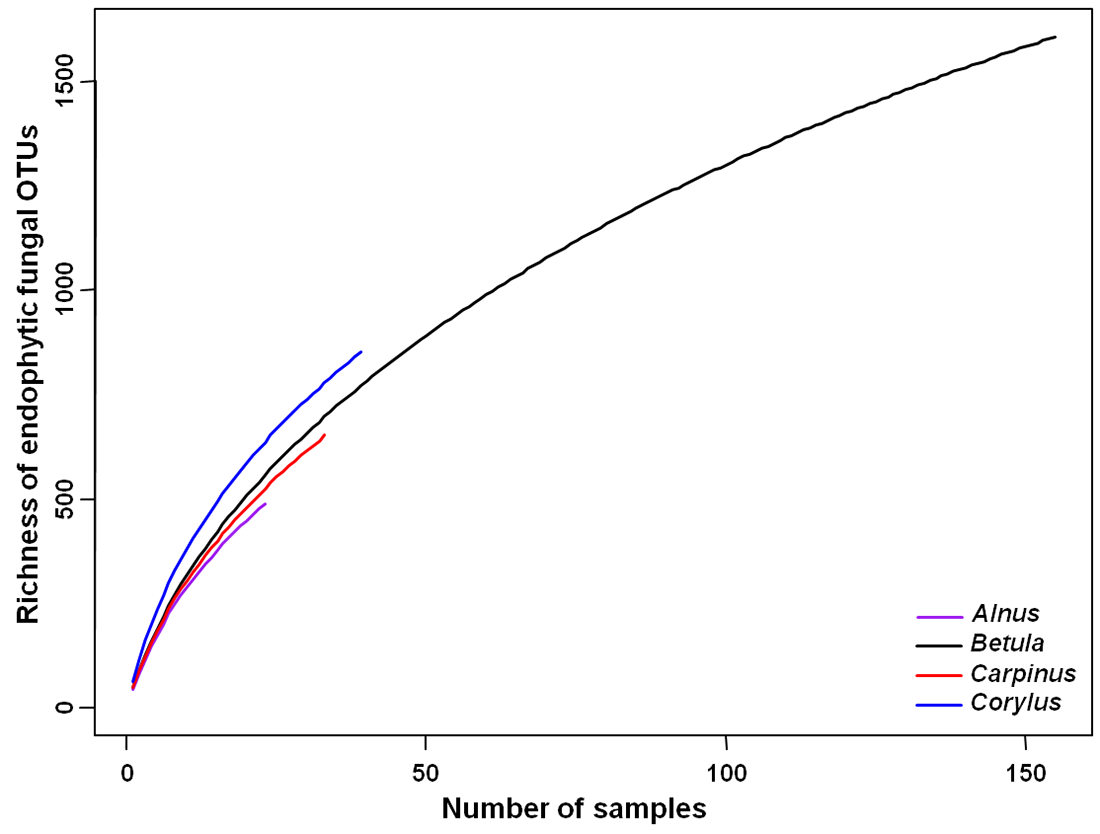
**

**Supplementary Figure 4.** Rarefaction of endophytic fungal operational taxonomic units (OTUs) in four plant genera.

## Supplementary Tables

**Supplementary Table 1.** Molecular identification of endophytic fungi investigated in this study. See excel file.

| **Supplementary Table 2.** All candidate models with ΔQAICc < 2 generated during model selection process. | | | | | |
| --- | --- | --- | --- | --- | --- |
| Candidate model | df | logLik | QAICc | ΔQAICc | Akaike weight |
| PCoA1+PCoA3+PCoA8+PCoA16+PCNM1+PCNM3+PCNM7+PCNM10+MAT | 10 | -1271.50 | 566.48 | 0.00 | 0.18 |
| PCoA1+PCoA3+PCoA8+PCoA16+PCNM1+PCNM2+PCNM3+PCNM7+PCNM10+MAT | 11 | -1268.19 | 567.28 | 0.80 | 0.12 |
| PCoA1+PCoA3+PCoA8+PCoA16+PCNM1+PCNM2+PCNM3+PCNM7+PCNM10+TC+MAT | 12 | -1263.79 | 567.62 | 1.14 | 0.10 |
| PCoA1+PCoA3+PCoA8+PCoA16+PCNM1+PCNM2+PCNM3+PCNM7+PCNM10+TN+MAT | 12 | -1264.62 | 567.98 | 1.49 | 0.08 |
| PCoA1+PCoA3+PCoA8+PCoA16+PCNM1+PCNM3+PCNM7+PCNM10+TN+MAT | 11 | -1269.86 | 567.99 | 1.51 | 0.08 |
| PCoA1+PCoA3+PCoA8+PCoA16+PCNM1+PCNM3+PCNM7+PCNM10+C:N+MAT | 11 | -1270.01 | 568.05 | 1.57 | 0.08 |
| PCoA1+PCoA3+PCoA8+PCoA16+PCNM1+PCNM3+PCNM7+PCNM10+TC+MAT | 11 | -1270.20 | 568.14 | 1.65 | 0.08 |
| PCoA1+PCoA3+PCoA8+PCoA16+PCNM1+PCNM3+PCNM7+PCNM10+MAT+MAP | 11 | -1270.43 | 568.24 | 1.75 | 0.07 |
| PCoA1+PCoA3+PCoA8+PCoA16+PCNM1+PCNM3+PCNM7+PCNM10+MAT+Altitude | 11 | -1270.57 | 568.30 | 1.81 | 0.07 |
| PCoA1+PCoA3+PCoA8+PCoA16+PCNM1+PCNM3+PCNM7+PCNM10+MAP | 10 | -1275.98 | 568.40 | 1.92 | 0.07 |
| PCoA1+PCoA3+PCoA8+PCoA16+PCNM1+PCNM3+PCNM7+PCNM10+TP+MAT | 11 | -1270.86 | 568.42 | 1.93 | 0.07 |
| PCoA, principal coordinates analysis of host phylogeny; PCNM, spatial principal coordinates of neighbor matrices; TC, total soil carbon; TN, total soil nitrogen; TP, total soil phosphorus; MAT, mean annual temperature; MAP, mean annual precipitation; QAICc, quasi-likelihood corrected Akaike information criterion; ΔAICc, the differences in QAICc between models. | | | | | |
